# The micronutrient content of the diet is correlated with serum glucose biomarkers and lipid profile and is associated with the odds of being overweight/obese—a case-control study

**DOI:** 10.3389/fnut.2023.1148183

**Published:** 2023-06-29

**Authors:** Farhad Vahid, Wena Rahmani, Sayed Hossein Davoodi, Torsten Bohn

**Affiliations:** ^1^Nutrition and Health Research Group, Department of Precision Health, Luxembourg Institute of Health, Strassen, Luxembourg; ^2^Nutrition Group, School of Health, Arak University of Medical Science, Arak, Iran; ^3^Department of Cellular and Molecular Nutrition, Faculty of Nutrition and Food Technology, Shahid Beheshti University of Medical Sciences, Tehran, Iran

**Keywords:** antioxidants, minerals, vitamins, body surface area (BSA), dietary assessment, insulin, public health, weight control

## Abstract

**Background:**

A low micronutrient intake has been reported to contribute to the double-burden of obesity, increasing the risk for chronic diseases such as cardiovascular disease, diabetes, cancer, and mental disorders. This case-control study compared micronutrient intake profiles in overweight/obese vs. normal-weight individuals. We hypothesized that a low intake of certain micronutrients would increase the odds of being overweight/obese.

**Methods:**

The case group (*n* = 812 adults) consisted of individuals with a BMI of ≥25  kg/m^2^, and the control group (*n* = 793) had BMIs of 17.9–24.9  kg/m^2^. A validated 124-item food frequency questionnaire was used to determine micronutrient-related dietary-quality, using the index of nutritional quality (INQ), calculated as the fraction of a micronutrient consumed vs. its dietary requirement. In addition, body surface area (BSA) was calculated according to the Mosteller formula.

**Results:**

The control group had significantly higher INQ-scores of vitamin A, vitamin C, calcium, magnesium, and selenium compared to the case group. Furthermore, individuals with normal BSA (≤1.91 m^2^ for men; ≤1.71  m^2^ for women) had significantly higher INQ scores of vitamin C, calcium, magnesium, and zinc compared to participants with high BSA. In multivariable adjustment regression models, INQs of vitamin C (OR_BMI_ = 0.79, 95%CI: 0.64–0.97; OR_BSA_ = 0.81, 95%CI, 0.68–0.97) and magnesium (OR_BMI_ = 0.69, 95%CI: 0.47–0.99; OR_BSA_ = 0.71, 95%CI: 0.52–0.97) were significantly associated with the odds of obesity/overweight (in both BMI and BSA categories).

**Conclusion:**

The significant association between micronutrient levels of the diet, especially of vitamin C and magnesium, with both obesity criteria, emphasized the importance of certain micronutrients in the obesity/overweight causal network.

## 1. Background

The prevalence of obesity and overweight in most countries is increasing rapidly, becoming a non-communicable pandemic (1, 2). Various factors, including genetics, gender, physical activity, economic/social/cultural/religious factors, as well as dietary factors, are intertwined with this pandemic (2, 3). The etiology of obesity is so complex and controversial that it is almost impossible to control or limit all of the contributing factors. On the other hand, obesity is strongly entangled with society’s health both directly and indirectly (2). Persons with obesity/overweight are at high risk for certain chronic diseases, including cardiovascular disease (CVD), type 2 diabetes (T2D), hypertension, various cancers, as well as mental disorders (4). In addition to the personal impact for concerned individuals, obesity/overweight imposes high costs on the countries’ health systems, both directly and by increasing the prevalence of co-morbidities such as non-communicable diseases (5). It should also be noted that even in communicable diseases, obese/overweight patients tend to be hospitalized longer, take more time to recover, respond later to treatment, or require more medication to recover, and they are also more likely to be re-infected (6–8). These factors drastically increase the treatment costs of obese/overweight people. On average, the annual treatment costs of a person with obesity are almost 30% higher than those of an individual with normal weight (9). Other additional costs, such as treating diseases for which obesity is a risk factor, are indirect and are not readily computable (9). However, dealing with persons with obesity in different countries and societies can be discriminatory, and these people often also suffer also psychologically (10).

Therefore, studying the various factors affecting the incidence and prevalence of obesity/overweight can be part of an effective strategy toward reducing the associated complications and consequences. From a nutritional point of view, various factors are influential regarding the incidence and prevalence of obesity/overweight. For example, several studies have examined the association between chrono-nutrition/meal timing (11, 12), macronutrient intake and their distribution ratio (13), calorie intake, as well as dietary patterns/behaviors (14), and micronutrient intake and the prevalence of obesity/overweight (3, 15).

Many studies and health associations, such as the World Health Organization (WHO), have highlighted the double burden of malnutrition of persons having obesity and micronutrient deficiency (16). Despite having a diet rich in energy and providing enough macronutrients—or even too many—many persons with overweight/obesity were reported to have low vitamin and mineral status due to marginal intake. For instance, obesity has often been associated with a low status of vitamin A (17), iron (18), zinc (19), and vitamin D status (20). Vitamins and minerals are involved in a huge array of metabolic processes, and reduced intake or status increases the risk of bodily dysfunctions (21). For example, low zinc status may compromise immune function as well as the efficiency of antioxidant enzymes such as catalase or superoxide dismutase (22) and has been related to decreased leptin levels (23) and, thus, satiety. Low intake of vitamin A has likewise been linked to increased obesity, perhaps due to its role in modulating RAR/RXR receptors that set the stage for many cellular developments and immune functions (24).

Thus, while many mechanisms and associations between micronutrients and overweight/obesity have been reported and documented, contradictions remain and require further investigation and documentation (1, 25–27). Our present study aimed at comparing the quality of the diet regarding micronutrient intake in individuals with obesity/overweight vs. normal-weight individuals. We hypothesized that a diet of lower nutrient density regarding micronutrients and decreased intake of vitamins and minerals would be related to increased odds of obesity/overweight.

## 2. Materials and methods

### 2.1. Participants

In this population-based case-control study, the case group [*n* = 812; overweight/obese individuals based on body mass index (BMI)] and the control group (*n* = 793; individuals with normal BMI) were selected from the patient’s caregivers attending Arak Medical Centers, Arak, Iran. First, we briefly explained the study’s objectives to the patient’s caregivers (who had come to visit the patients), and if persons were interested in joining the study, their height and weight were measured. Based on the calculated BMIs, participants were allocated into case groups (overweight/obese) or controls (normal weight). However, we additionally categorized them in statistical analyses based on the body surface area (BSA) after considering inclusion and exclusion criteria.

Following the completion of the questionnaires, participants were referred to the specified accredited laboratories for biological tests. The case group consisted of individuals with a higher or equal BMI of 25 kg/m^2^, and the control group had a BMI of 17.9–24.9 kg/m^2^. The cases and controls were frequently matched in terms of age and gender. In this regard, 50.4% (*n* = 409) of the case group and 50.2% (*n* = 398) of the control group were women. The mean age was 47.6 ± 13.0 years in the case group and 48.4 ± 12.7 years in the control group. Written informed consent was received from all participants, and the Arak University of Medical Science Ethics Committee, Arak, Iran, approved the study protocol (Ethics Committee No. IR.ARAKMU.REC.1398.094). The study protocol and method have been published elsewhere (28, 29).

#### 2.1.1. Inclusion and exclusion criteria

The inclusion were: (a) Lack of active diseases such as CVD and cancer; (b) Not following a special diet such as vegetarian; (c) No major change in diet in the past year; (d) No pregnancy or lactation; (e) No medication or dietary supplements in the past 6 months; (f) Willingness to participate in the study; (g) Absence of drug addiction; (k) Living in Arak for the past 5 years, I. Being in the age range of 18–81 years. An exclusion criteria was reporting less than 80% of the questions in the questionnaire.

#### 2.1.2. Assessment of dietary intake

A validated and reliable 124-item food frequency questionnaire (FFQ), including commonly consumed Iranian food items, was used to assess dietary intake (30). Individuals were asked to report the amount and frequency of food and beverage consumption in the previous year. Frequency of use was reported as “never use,” “daily,” “weekly,” “monthly” or “annually.” The “daily” or “weekly” intake in the season in question was asked for seasonal food items. Food intake was evaluated by means of the Nutritionist ІV software (First Databank, Hearst Corp., San Bruno, CA, United States), and the daily average of macro- and micronutrients was extracted. The total energy was obtained as the sum of 9 kcal/g for fat, 4 kcal/g for protein, 4 kcal/g for carbohydrates, and 2 kcal/g for dietary fiber. Daily intake was aggregated as a total of food in 1 year and divided by 365 to obtain the average daily intake.

During several sessions, a nutritionist provided the training to complete the questionnaires accurately to medical staff. All questionnaires were completed in person by the trained medical staff.

#### 2.1.3. Assessment of blood tests

After 8–12 h of fasting, 5 mL of intravenous blood was obtained from all participants. Serum was then collected following centrifugation (2,000 g for 10 min) and stored at −80°C until further analyses. Blood glucose biomarkers and lipid profiles were measured by approved laboratories using the following methods.

According to the kit instructions, fasting blood glucose (FBG, in mg/dL), insulin (mIU/mL), glycated hemoglobin (HbA1C, in mmol/mol), low-density lipoprotein cholesterol (LDL-C, in mg/dL), triglycerides (TG, in mg/dL), high-density lipoprotein cholesterol (HDL-C, in mg/dL), total cholesterol (in mg/dL) were assessed using relevant commercial ELISA kits (all provided by Pishtazteb Co.; Tehran, Iran), following the manufacturer’s guidelines.

#### 2.1.4. Assessment of anthropometric and socioeconomic variables

For all participants, BMI was obtained by dividing squared height (meters) by weight (kg). Seca scales (Seca; Hamburg, Germany) measured the individuals’ weight with an accuracy of 50 g, and the height was assessed using a tape measure attached to the wall with an accuracy of 1 cm. The BMI was considered “normal” if the value was between 17.9 and 24.9 kg/m^2^, and obese/overweight if the value was equal to or higher than 25 kg/m^2^. Body surface area (BSA) was calculated according to the Mosteller formula: BSA = (height (cm) * weight (kg)/3,600)^1/2^ (31). The BSA was considered ‘normal’ if the mean value was ≤1.91 m^2^ for men and ≤1.71 m^2^ for women.

A general information questionnaire collected information on age (year), gender (female/male), marital status (single/married/widow, divorced, separated, not willing to mention, living with a partner), education (lower than high school diploma/higher than diploma), regular physical activity (yes/no), smoking (yes/no), alcohol (yes/no), history of CVD (yes/no), T2D (yes/no), and hypertension (yes/no).

#### 2.1.5. Assessment of dietary quality regarding micronutrient intake

We estimated micronutrient quality using each micronutrient’s index of nutritional quality (INQ) for vitamins A, C, D, E, K, thiamin, riboflavin, niacin, biotin, folate, pantothenic acid, B12, and B6 and calcium, iron, magnesium, zinc, copper, selenium, and manganese. For calculating INQs of micronutrients in which existed a defined recommended dietary allowance (RDA) or adequate intake (AI) in dietary reference intake (DRI) tables, we used the following formula: INQ = (consumed amount of a micronutrient per 1,000 kcal)/(RDA or AI of that micronutrient per 1,000 kcal) (19, 20, 23). FFQ-derived dietary data were used to calculate INQ scores.

### 2.2. Statistical analysis

Original power calculations were based on an assumed *p*-value of 0.05 (alpha), a targeted power of 0.80, and an assumed P_0_ (probability of exposure in controls), and P_1_ (probability of exposure in case subjects) or odds ratio, based on literature findings.

Q-Q normality plots and the Kolmogorov–Smirnov (KS) test were employed to verify the normality of the variables’ distribution. For non-normal distributed variables, a log-transformation was performed (log-transferred data were used in all analyses). The INQs were investigated across the following characteristics of participants (both BMI and BSA groups): age, gender, education, smoking, alcohol, marital status, regular physical activity, history of CVD, T2D, and hypertension analyzed by independent *t*-tests or chi-squared (*χ*^2^) tests for continuous and categorical variables, respectively. Odds ratio (OR) and 95% confidence intervals (CI) for obesity/overweight using BMI and also BSA as outcomes were estimated using logistic regression in three models. In model A, crude ORs and 95% CIs were reported. In model B, we adjusted for age and gender. Model C was model B+ with additional adjustments for education, smoking, alcohol, marital status, regular physical activity, history of CVD, T2D, and hypertension. Bivariate and partial correlation were used to investigate the correlation between INQs and blood glucose handling markers and lipid profile. Partial correlation was done by controlling for age, gender, education, smoking, alcohol, marital status, regular physical activity, history of CVD, T2D, and hypertension. All statistical analyses were performed using SAS^®^ 9.3 (SAS Institute Inc., Cary, NC); all *p*-values were based on two-sided tests. A *p*-value below 0.05 (2-sided) was considered statistically significant. Data reported in the descriptive analyses represent mean and standard deviation (SD) or number (percentage).

## 3. Results

The distribution of participants’ characteristics across BMI groups is represented in Table 1. There was a significant difference in the history of the disease. Participants with obesity/overweight reported a higher prevalence of a history of CVD, T2D, and hypertension compared to normal-weight individuals (all *p*-values < 0.01). In addition, there was a significant difference between blood glucose handling biomarkers and lipid profile between cases and controls. Individuals with obesity/overweight had a significantly higher level of FBG, insulin, HbA1C, LDL-C, TG, and total cholesterol compared to normal-weight participants. On the contrary, the control group exhibited a significantly higher level of HDL-C compared to the case group.

**Table 1:** Distribution of characteristics across BMI groups of participants of the case-control study^f^.

Characteristics	Mean ± SD or number (%)			*p*-value^a^
	Cases (*n* = 812)	Controls (*n* = 793)	Total (*n* = 1,605)	
Age (years)	47.6 ± 13.0	48.4 ± 12.7	48.0 ± 12.9	0.26
BMI (kg/m^2^)	27.8 ± 2.5	22.2 ± 1.7	25.0 ± 3.5	**<0.01**
FBG (mg/dL)	94.2 ± 21.0	92.1 ± 18.1	93.2 ± 19.6	**0.03**
Insulin (mIU/mL)	8.1 ± 11.9	7.2 ± 4.6	7.7 ± 9.0	**0.04**
HbA1C (mmol/mol)	18.8 ± 15.5	15.1 ± 12.2	17.0 ± 14.1	**<0.01**
LDL-C (mg/dL)	129.9 ± 34.6	126.5 ± 33.4	128.2 ± 34.1	**0.05**
TG (mg/dL)	128.5 ± 77.8	117.0 ± 88.1	122.8 ± 83.2	**<0.01**
HDL-C (mg/dL)	52.1 ± 14.0	56.0 ± 14.2	54.0 ± 14.2	**<0.01**
Cholesterol (mg/dL)	202.4 ± 38.3	198.1 ± 36.2	200.3 ± 37.4	**0.02**
Gender:				0.96
Women	409 (50.3%)	398 (50.2%)		
Men	403 (49.7%)	395 (49.8%)		
Education:				0.71
Diploma and low literate	545 (67.1%)	525 (66.2%)		
Higher than diploma	267 (32.8%)	268 (33.8%)		
Smoking status:				0.99
Non-smokers	680 (83.7%)	664 (83.7%)		
Alcohol consumption				0.75
No	723 (89.0%)	702 (88.5%)		
Marital status:				0.91
Married	615 (75.7%)	600 (75.6%)		
Single	136 (16.7%)	137 (17.2%)		
Other	61 (7.6%)	56 (7.2%)		
Regular physical activity:				0.06
No	628 (77.4%)	580 (73.2%)		
T2D:				**<0.01**
Yes	246 (30.3%)	190 (24.0%)		
CVD:				**<0.01**
Yes	204 (25.1%)	109 (13.7%)		
Hypertension:				**<0.01**
Yes	495 (61.0%)	332 (41.8%)		

f A BMI equal to or higher than 25 kg/m^2^ was considered obese/overweight and between 17.9 and 24.9 kg/m^2^ as normal weight.

a Independent Student’s *t*-test was used for comparing continuous variables, and a Chi-square test was used for categorical variables; significant *p*-values are shown in bold.

SD, Standard deviation; BMI, Body mass index; HbA1C, Glycated hemoglobin; LDL-C, Low-density lipoprotein cholesterol; TG, Triglycerides; HDL-C, High-density lipoprotein cholesterol; T2D, Type 2 diabetes; CVD, Cardiovascular diseases.

A comparison of the INQs of the participants based on BMI groups is represented in Table 2. Controls (normal BMI) had a significantly higher INQ for vitamin A, vitamin C, calcium, magnesium, and selenium compared to the case group.

**Table 2:** Comparison of the participants’ index of nutritional quality (INQ) regarding micronutrient intake patterns based on BMI groups^f^.

INQs	Mean ± SD			RDA (or AI) Women-Men	*p*-value^a^
	Cases (*n* = 812)	Controls (*n* = 793)	Total (*n* = 1,605)		
Vitamin A	0.56 ± 0.29	0.60 ± 0.31	0.58 ± 0.30	700–900 µg/d (RAE/d)	**0.02**
Vitamin D	0.10 ± 0.07	0.10 ± 0.08	0.10 ± 0.08	15–15 µg/d	0.66
Vitamin E	0.89 ± 0.41	0.91 ± 0.43	0.90 ± 0.42	15–15 mg/d	0.32
Vitamin K	1.88 ± 1.00	1.93 ± 1.05	1.91 ± 1.03	90–120 µg/d	0.37
Vitamin C	1.29 ± 0.58	1.38 ± 0.64	1.33 ± 0.61	75–90 mg/d	**<0.01**
Thiamin	1.36 ± 0.62	1.39 ± 0.64	1.38 ± 0.63	1.1–1.2 mg/d	0.43
Riboflavin	1.27 ± 0.54	1.31 ± 0.56	1.29 ± 0.55	1.1–1.3 mg/d	0.16
Niacin	1.39 ± 0.59	1.42 ± 0.61	1.40 ± 0.60	14–16 mg/d	0.26
Vitamin B6	1.14 ± 0.45	1.16 ± 0.48	1.15 ± 0.47	1.3–1.7 mg/d	0.32
Folate	1.21 ± 0.51	1.25 ± 0.51	1.23 ± 0.51	400–400 µg/d	0.12
Vitamin B12	1.62 ± 1.00	1.68 ± 1.04	1.65 ± 1.02	2.4–2.4 µg/d	0.25
Biotin	0.89 ± 0.34	0.91 ± 0.35	0.90 ± 0.35	30–30 µg/d	0.27
Pantothenic acid	1.07 ± 0.45	1.09 ± 0.45	1.08 ± 0.45	5–5 mg/d	0.32
Calcium	0.75 ± 0.31	0.80 ± 0.32	0.77 ± 0.31	1,000–1,000 mg/d	**<0.01**
Iron	1.67 ± 0.62	1.71 ± 0.66	1.70 ± 0.64	18–8 mg/d	0.14
Magnesium	0.99 ± 0.32	1.03 ± 0.35	1.01 ± 0.34	320–420 mg/d	**0.03**
Zinc	1.10 ± 0.42	1.14 ± 0.45	1.12 ± 0.43	8–11 mg/d	0.09
Copper	2.08 ± 1.05	2.12 ± 1.08	2.10 ± 1.07	900–900 µg/d	0.44
Manganese	3.09 ± 1.34	3.18 ± 1.38	3.13 ± 1.36	1.8–2.3 mg/d	0.18
Selenium	1.61 ± 0.63	1.72 ± 0.77	1.66 ± 0.70	55–55 µg/d	**<0.01**

f A BMI equal to or higher than 25 kg/m^2^ was considered obese/overweight and between 17.9 and 24.9 kg/m^2^ as normal.

a Independent sample *t*-test was used for comparing INQs; significant *p*-values are shown in bold. RAE: retionoic acid equivelents.

Table 3 represents a comparison of the INQs of the participants based on BSA groups. Individuals with normal BSA (different categories were considered for gender) had significantly higher INQ of vitamin C, calcium, magnesium, and zinc compared to participants with high BSA. Comparing the results of Tables 2, 3 revealed that only INQ of vitamin C, calcium, and magnesium differed significantly between the controls (normal BMI or normal BSA) and cases in both categories (BMI and BSA categories).

**Table 3:** Comparison of the participants’ index of nutritional quality (INQ) regarding micronutrient intake patterns based on BSA groups^f^.

INQs	Mean ± SD		*p*-value^a^
	Cases (*n* = 818)	Controls (*n* = 787)	
Vitamin A	0.58 ± 0.30	0.59 ± 0.30	0.65
Vitamin D	0.10 ± 0.07	0.11 ± 0.08	0.06
Vitamin E	0.89 ± 0.42	0.91 ± 0.43	0.25
Vitamin K	1.87 ± 1.02	1.95 ± 1.04	0.14
Vitamin C	1.30 ± 0.57	1.37 ± 0.64	**0.02**
Thiamin	1.38 ± 0.60	1.38 ± 0.66	0.94
Riboflavin	1.28 ± 0.54	1.30 ± 0.55	0.30
Niacin	1.39 ± 0.60	1.41 ± 0.61	0.53
Vitamin B6	1.13 ± 0.47	1.17 ± 0.46	0.13
Folate	1.21 ± 0.50	1.24 ± 0.52	0.16
Vitamin B12	1.65 ± 1.01	1.65 ± 1.03	0.96
Biotin	0.89 ± 0.35	0.90 ± 0.35	0.60
Pantothenic acid	1.07 ± 0.44	1.08 ± 0.47	0.53
Calcium	0.76 ± 0.31	0.79 ± 0.32	**0.04**
Iron	1.70 ± 0.63	1.68 ± 0.65	0.44
Magnesium	0.99 ± 0.32	1.03 ± 0.35	**<0.01**
Zinc	1.09 ± 0.40	1.15 ± 0.46	**<0.01**
Copper	2.09 ± 1.04	2.12 ± 1.10	0.48
Manganese	3.08 ± 1.35	3.19 ± 1.37	0.09
Selenium	1.66 ± 0.67	1.67 ± 0.74	0.85

f The BSA was considered “normal” if the mean value was ≤1.91 m^2^ for men and ≤1.71 m^2^ for women.

a Independent sample *t*-test was used for comparing INQs; significant *p*-values are shown in bold.

Table 4 represents ORs and CIs for the association between INQs and BMI groups. The INQ of vitamin A, C, calcium, magnesium, and selenium as continuous variables in all three models (crude model, age and gender-adjusted model, and multivariable-adjusted model) was significantly associated with BMI. The INQ of iron, however, was only significantly associated with BMI in the age and gender (model 2) as well as the fully adjusted model (model 3).

**Table 4:** Odds ratios (ORs) and 95% confidence intervals (CIs) for the association between INQ and BMI groups^f^.

INQs^ꝉ^	Crude model		Adjusted model 1		Adjusted model 2	
	ORs and CI 95%^a^	*p*-value	ORs and CI 95%^b^	*p*-value	ORs and CI 95%^c^	*p*-value
Vitamin A	**0.68 (0.49–0.94)**	**0.02**	**0.58 (0.39–0.87)**	**<0.01**	**0.59 (0.39–0.89)**	**0.01**
Vitamin D	0.76 (0.22–2.64)	0.66	1.10 (0.24–5.07)	0.89	1.19 (0.24–5.73)	0.82
Vitamin E	0.89 (0.70–1.12)	0.32	0.87 (0.65–1.16)	0.34	0.85 (0.63–1.15)	0.30
Vitamin K	0.95 (0.87–1.05)	0.37	0.95 (0.84–1.07)	0.38	0.96 (0.85–1.08)	0.49
Vitamin C	**0.78 (0.66–0.92)**	**<0.01**	**0.77 (0.63–0.94)**	**0.01**	**0.79 (0.64–0.97)**	**0.03**
Thiamin	0.94 (0.80–1.09)	0.43	0.91 (0.75–1.11)	0.38	0.89 (0.73–1.09)	0.28
Riboflavin	0.88 (0.73–1.05)	0.16	0.91 (0.73–1.14)	0.43	0.90 (0.72–1.14)	0.41
Niacin	0.91 (0.77–1.07)	0.26	0.84 (0.69–1.03)	0.09	0.85 (0.69–1.04)	0.11
Vitamin B6	0.90 (0.73–1.11)	0.32	0.95 (0.74–1.22)	0.71	1.01 (0.77–1.31)	0.93
Folate	0.86 (0.71–1.04)	0.12	0.89 (0.70–1.13)	0.36	0.90 (0.70–1.15)	0.41
Vitamin B12	0.94 (0.86–1.04)	0.25	0.94 (0.84–1.06)	0.32	0.93 (0.83–1.05)	0.30
Biotin	0.85 (0.65–1.12)	0.27	0.93 (0.66–1.31)	0.68	0.92 (0.64–1.30)	0.64
Pantothenic acid	0.89 (0.72–1.11)	0.32	0.86 (0.66–1.11)	0.26	0.84 (0.64–1.10)	0.21
Calcium	**0.62 (0.45–0.84)**	**<0.01**	**0.58 (0.40–0.85)**	**<0.01**	**0.59 (0.40–0.87)**	**<0.01**
Iron	0.89 (0.76–1.04)	0.14	**0.78 (0.64–0.95)**	**0.01**	**0.79 (0.65–0.96)**	**0.02**
Magnesium	**0.72 (0.54–0.97)**	**0.03**	**0.69 (0.48–0.98)**	**0.04**	**0.69 (0.47–0.99)**	**0.05**
Zinc	0.82 (0.66–1.03)	0.09	0.90 (0.68–1.19)	0.48	0.94 (0.70–1.25)	0.66
Copper	0.96 (0.88–1.05)	0.44	0.98 (0.88–1.10)	0.80	0.99 (0.88–1.11)	0.88
Selenium	**0.79 (0.69–0.91)**	**<0.01**	**0.71 (0.60–0.85)**	**<0.01**	**0.70 (0.58–0.84)**	**<0.01**
Manganese	0.95 (0.88–1.02)	0.18	0.97 (0.89–1.06)	0.50	0.96 (0.88–1.05)	0.43

a Crude model.

b Model A: Age and sex-adjusted.

c Model B: Model A+ Education, smoking, alcohol, marital status, regular physical activity, history of CVD, type 2 diabetes, and hypertension adjusted.

f The BMI equal to or higher than 25 kg/m^2^ was considered obese/overweight and between 17.9 and 24.9 kg/m^2^ as normal weight.

ꝉ All values are based on log-transformed data; significant *p*-values are shown in bold.

Table 5 shows ORs and CIs for the association between INQ and BSA groups. The INQ of vitamin C, magnesium, and zinc as continuous variables in all three models (crude model, age and gender-adjusted model, and multivariable-adjusted model) was significantly associated with BSA. The INQ of calcium was only significantly associated with BSA in the crude model. Comparing the results of Tables 4, 5 revealed that only the INQ of vitamin C and magnesium were significantly associated with both categories of obesity (BMI and BSA categories) in the fully adjusted model.

**Table 5:** Odds ratios (ORs) and 95% confidence intervals (CIs) for the association between INQ and BSA groups^f^.

	Crude model		Adjusted model 1		Adjusted model 2	
INQs	ORs and CI 95%^a^	*p*-value	ORs and CI 95%^b^	*p*-value	ORs and CI 95%^c^	*p*-value
Vitamin A	0.93 (0.67–1.28)	0.65	1.01 (0.71–1.43)	0.95	1.06 (0.75–1.50)	0.72
Vitamin D	0.30 (0.08–1.04)	0.06	0.41 (0.10–1.56)	0.19	0.46 (0.12–1.80)	0.26
Vitamin E	0.87 (0.69–1.10)	0.25	0.98 (0.77–1.26)	0.92	1.02 (0.79–1.31)	0.87
Vitamin K	0.93 (0.84–1.02)	0.14	0.92 (0.83–1.01)	0.10	0.91 (0.82–1.02)	0.11
Vitamin C	**0.83 (0.70–0.97)**	**0.02**	**0.80 (0.67–0.95)**	**0.01**	**0.81 (0.68–0.97)**	**0.02**
Thiamin	0.99 (0.85–1.16)	0.94	1.02 (0.87–1.21)	0.76	1.02 (0.87–1.21)	0.75
Riboflavin	0.91 (0.76–1.08)	0.30	0.93 (0.77–1.12)	0.46	0.95 (0.78–1.15)	0.63
Niacin	0.95 (0.80–1.11)	0.53	0.94 (0.79–1.12)	0.53	0.96 (0.81–1.15)	0.70
Vitamin B6	0.85 (0.69–1.05)	0.14	0.91 (0.73–1.14)	0.42	0.96 (0.77–1.21)	0.76
Folate	0.87 (0.72–1.06)	0.16	0.90 (0.73–1.10)	0.31	0.91 (0.74–1.12)	0.40
Vitamin B12	0.99 (0.90–1.01)	0.96	1.00 (0.90–1.11)	0.99	1.00 (0.91–1.11)	0.90
Biotin	0.93 (0.70–1.22)	0.60	0.91 (0.68–1.23)	0.57	0.93 (0.69–1.26)	0.66
Pantothenic acid	0.93 (0.75–1.15)	0.53	0.94 (0.75–1.19)	0.64	0.97 (0.77–1.23)	0.81
Calcium	**0.73 (0.54–0.99)**	**0.05**	0.75 (0.54–1.05)	0.09	0.79 (0.57–1.11)	0.17
Iron	1.06 (0.91–1.23)	0.44	1.06 (0.90–1.25)	0.43	1.08 (0.91–1.27)	0.36
Magnesium	**0.67 (0.51–0.92)**	**0.01**	**0.70 (0.51–0.95)**	**0.02**	**0.71 (0.52–0.97)**	**0.04**
Zinc	**0.75 (0.60–0.94)**	**0.01**	**0.68 (0.53–0.87)**	**<0.01**	**0.70 (0.55–0.89)**	**<0.01**
Copper	0.97 (0.88–1.06)	0.48	0.96 (0.87–1.05)	0.40	0.96 (0.87–1.06)	0.46
Selenium	0.98 (0.86–1.13)	0.85	0.97 (0.83–1.12)	0.68	0.99 (0.85–1.15)	0.91
Manganese	0.94 (0.87–1.01)	0.09	0.93 (0.86–1.01)	0.08	0.94 (0.87–1.01)	0.12

a Crude model.

b Model A: Age and sex-adjusted.

c Model B: Model A+ education, smoking, alcohol, marital status, regular physical activity, history of CVD, type 2 diabetes, and hypertension adjusted.

f The BSA was considered “normal” if the mean value was ≤1.91 m^2^ for men and ≤1.71 m^2^ for women.

Significant *p*-values are shown in bold.

INQ, Index of nutritional quality; BSA, Body surface area.

In addition, we performed sensitivity analyses based on gender groups for the fully adjusted model considering BMI and BSA. The sensitivity analysis results were in line with the main results, although the number of significant associations was higher in men than in women (Supplementary Table S1).

**Figure 1:**
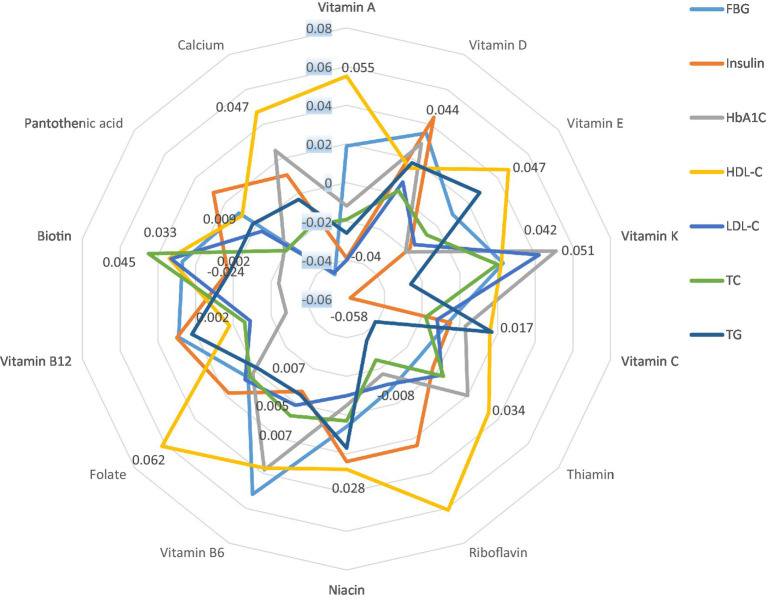
Partial correlation* between INQs and blood glucose handling markers and lipid profile controlling for age, gender, education, smoking, alcohol, marital status, regular physical activity, history of CVD, type 2 diabetes, and hypertension. INQ, Index of nutritional quality; FBG, Fasting blood glucose; HbA1C, Glycated hemoglobin; LDL-C, Low-density lipoprotein cholesterol; TC, Total cholesterol; TG, Triglycerides; HDL-C, High-density lipoprotein cholesterol. * Only significant values are shown. Results regarding micronutrients that had no significant correlation with any of the indicators and bivariate correlation models are shown in Supplementary Table S1.

Finally, Figure 1 represents the partial correlation controlling for age, gender, education, smoking, alcohol, marital status, regular physical activity, history of CVD, T2D, and hypertension between INQs and blood glucose handling markers and lipid profile. In the partial model, there was a significant correlation between the INQ of vitamin A and HDL-C and LDL-C; calcium and FBS, HDL-C, LDL-C; iron and HDL-C; vitamin D and insulin, riboflavin and HDL-C; vitamin B6 and FBS; folate and HDL-C; vitamin K and insulin, HbA1C; magnesium and HbA1C, LDL-C, total cholesterol, and triglycerides; zinc and HDL-C; manganese and HDL-C. Similar results were obtained in bivariate correlation models (Supplementary Table S2).

## 4. Discussion

In studying the dietary patterns regarding micronutrient adequacy based on BMI grouping, a significantly lower score of the INQ was found for vitamins A and C, calcium, magnesium, and selenium of cases vs. controls. Similarly, based on the BSA grouping, a significant difference was observed between the INQ of vitamin C, calcium, magnesium, and zinc between cases and controls; and vitamin C, calcium, and magnesium were found to differ based on both categories. Our study, based on multivariate-adjusted models, showed significantly higher INQs of vitamin A, C, calcium, magnesium, and selenium, which were inversely related to overweight/obesity based on BMI, and also a lower INQ for vitamin C, magnesium, and zinc in the overweight/obesity group based on and BSA, with only vitamin C and magnesium being significantly inversely associated with both classifications.

The association between INQ and obesity/overweight (based on BMI) has been studied in two previous studies (3, 32). Gholamalizadeh et al. (3) examined the association between the risk of obesity and INQ in adolescent boys. They reported a significant inverse association between the INQ of vitamin C, iron, vitamin B6, pantothenic acid, selenium, and magnesium and the risk of obesity in adolescent boys (3). They also reported that after adding different confounders to the model, a significant inverse relationship, in addition to the above, was observed between the INQ of zinc and obesity risk (3).

Mehrdad et al. (32) investigated the relationship between INQ and obesity in adults. Similar to the present study, their results emphasized an inverse association between the INQ of vitamin D and manganese and obesity. The association between vitamin D intake and obesity has been shown in other studies (33–35) and may be explained by the importance of this vitamin for the immune system and systemic inflammation and oxidative stress that are aggravating factors in obesity (36, 37). In the present study, vitamin D intake was similar, though rather quite low in both groups, a fact that had been previously reported for the region (3). However, none of the two studies (3, 32) have investigated the relationship between the quality of micronutrients and “obesity” based on the BSA, so it is impossible to compare results in this regard.

The finding that vitamin C was significantly associated with obesity in our study (based on BMI and BSA) and also by Gholamalizadeh et al. (3) maybe is due to its antioxidant and, thus, indirect anti-inflammatory effect (38–40). In fact, studies have shown that people exposed to a high amount of reactive oxygen species (ROS) are more prone to being obese or overweight (41). However, it should also be noted that obesity itself can result in increased production of ROS (41). In addition, vitamin C is a good indicator of fruit and vegetable intake in general (42), and thus its consumption may reflect an altogether more healthy dietary pattern that also contains more dietary fiber and also secondary plant metabolites such as polyphenols and carotenoids that also appear to play some protective roles against obesity (43–45).

Although our study was not able to examine the possible mechanisms underlying the role of vitamin C and obesity, its antioxidant properties have been reported to protect to a certain degree from inflammation and oxidative stress (38–40). On the other hand, persons in this study, including the cases, consumed 25–30% more vitamin C than the RDA and can thus be judged as having an adequate vitamin C status. Nevertheless, the evidence remains controversial, and taking vitamin C supplements does not appear to prevent obesity or being overweight (46), especially if persons are not deficient in this vitamin. However, recommending foods rich in vitamin C can be an effective strategy to improve inflammatory and oxidative stress status and decrease CVD risk in people with obesity/overweight (47, 48).

In addition, the role of magnesium—having a significant association with overweight/obesity with both BMI and BSA—in preventing atherosclerosis, and stroke, lowering blood pressure, cholesterol, and triglycerides, correcting irregular heartbeat, and reducing insulin requirements in diabetic individuals has been well demonstrated (49, 50). Although the mechanisms of its direct effects in preventing or controlling obesity are controversial, long-term low magnesium intake has been associated with increased insulin resistance, leading to a cascading network of factors that eventually might lead to obesity or overweight (51, 52). Magnesium participates in a large number of energy-related enzymatic reactions (53) and has been shown to potentially reduce blood pressure, hypertriglyceridemia, and hyperglycemia (54). It also has been emphasized that its intake has rather globally declined during the last decades (55). Although the mentioned mechanisms may partially explain the relationship between low magnesium intake and obesity, they cannot justify the whole causal network (56). Especially as in the present study, most persons appeared to reach the RDA, and a deficiency is therefore not likely.

The advantages of using INQs are that it considers nutrient intake based on total calorie intake and compares them with the RDA (57–59). As a result, it is expected that the obtained results will be more realistic than the study of merely the amount of micronutrient intake. However, it should be emphasized that studies with longitudinal designs with appropriate sample sizes are needed to confirm the sensitivity and specificity of the INQ as well as its applicability in nutritional studies.

One of the important strengths of our study is investigating the correlation between INQ and blood-based biomarkers. While remarkably, vitamin C was not associated with any measured biomarker, magnesium was related to decreased HbA1C, total cholesterol, and LDL-C. Earlier studies had already emphasized that persons with a better magnesium status tended to show improvements in glucose control and blood lipids (60, 61). We can only speculate on the absence of a relation of vitamin C and any measurable effects. It is possible that the action of vitamin C rather influenced markers of inflammation and oxidative stress, which were not assessed in the present study, and that differences in vitamin C were not pronounced enough to produce changes in the observed biomarkers of blood lipids and glucose control.

Therefore, this investigation allowed us to measure the predictive power of INQ. Furthermore, one of the limitations of nutritional studies is the lack of attention to discussing the calibration of the studied methods, i.e., that the results obtained may be significant but, in fact, have little or no clinical importance or significance. Therefore, the results of our study, in addition to being significant, could also point toward clinical importance. Another strength of the study was using a valid and reliable FFQ. This allowed us to have a comprehensive and complete overview of micronutrient intake. In addition, a population-based case-control study design allowed us to control for a wide range of variables and confounders. However, due to budget limitations, we had to disregard some potential confounding factors, such as genetic differences.

Thus, disregarding genetic differences was one of the major limitations of our study. However, as the study was conducted with a relatively appropriate sample size in a population-based design, following age and gender-matched design, it can be concluded that the results can be generalized to the majority of the population living under similar conditions. Another limitation of our study was using FFQ, which is prone to recall bias. However, trained personnel completed the FFQs, and also, because the questionnaire had already been validated in previous studies, it seems that this bias would not seriously harm the results. Another limitation of our study was the lack of body composition indicators such as body fat or fat-free tissue percentage. Budget restrictions and lack of access to more sophisticated devices such as DEXA were the main reasons for this limitation. However, as one of our important strengths, we used two different definitions of obesity (BMI and BSA) to minimize this limitation, although we recommend that future studies consider body composition for more accurate results.

After considering strengths and limitations, potentials of the present study include revealing how the intake of specific micronutrients affects glucose biomarkers and lipid profile levels in individuals. Understanding these associations can provide valuable insights into the role of micronutrients in metabolic health. By comparing the micronutrient intake of overweight/obese individuals with that of non-overweight individuals (controls), this study can establish a potential link between micronutrient intake and weight status. This can contribute to developing targeted interventions for obesity prevention and management. Thus, the ultimate goal of this study was to provide evidence-based recommendations for improving metabolic health and reducing the risk of overweight/obesity.

Finally, the predominant limitation of this study was that as a case-control study, the design can identify associations but cannot determine causality. Various confounding factors, such as overall diet quality, physical activity levels, and genetic predispositions, may influence the observed relationships. Addressing these confounding factors and considering potential biases are crucial to strengthen the study’s findings. Additionally, accurately assessing dietary intake can be challenging due to reliance on self-reported data, recall bias, and variations in portion sizes and food composition. Overcoming these challenges requires meticulous study design, robust data collection methods, and appropriate statistical analyses.

## 5. Conclusion

The results of our study add to the evidence of the role of dietary micronutrient adequacy in people with overweight and obesity, in line with the double burden of malnutrition. The association observed between INQ of vitamin C and magnesium with respect to both obesity criteria (BMI and BSA) highlights the importance of these two micronutrients. Possible mechanisms need to be further explored and should be followed in the following cohort-design studies. In conclusion, our study highlighted the role of dietary adequacy regarding micronutrient intake in association with overweight/obesity and considers the use of the INQ to be preferable to the use of micronutrient intake alone to assess nutritional status. In addition, considering different definitions (classifications) of obesity may affect the results; therefore, considering the most suitable methods of defining obesity, including body composition analysis along with the more traditional BMI or BSA, would be necessary.

## Data availability statement

The data presented in this study are available on request from the corresponding author. Due to our institute’s rules and laws, the data are not publicly available.

## Ethics statement

The studies involving human participants were reviewed and approved by Arak University of Medical Science Ethics Committee, Arak, Iran, approved the study protocol (Ethics Committee No. IR.ARAKMU.REC.1398.094). The patients/participants provided their written informed consent to participate in this study.

## Author contributions

FV designed the study, performed the statistical analyses, and interpreted the data. WR was involved in the data collection. FV and SHD drafted the manuscript. TB provided expertise and oversight on the intellectual content. All authors contributed to the article and approved the submitted version.

## Conflict of interest

The authors declare that the research was conducted in the absence of any commercial or financial relationships that could be construed as a potential conflict of interest.

## Publisher’s note

All claims expressed in this article are solely those of the authors and do not necessarily represent those of their affiliated organizations, or those of the publisher, the editors and the reviewers. Any product that may be evaluated in this article, or claim that may be made by its manufacturer, is not guaranteed or endorsed by the publisher.

## Figures and Tables

**Table:** 

CVD	Cardiovascular Disease
T2D	Type 2 Diabetes
WHO	World Health Organization
BMI	Body Mass Index
BSA	Body Surface Are
FFQ	Food Frequency Questionnaire
FBG	Fasting Blood Glucose
HbA1C	Glycated Hemoglobin
LDL-C	Low-Density Lipoprotein Cholesterol
TG	Triglycerides
HDL-C	High-Density Lipoprotein Cholesterol
INQ	Index of Nutritional Quality
RDA	Recommended Dietary Allowance
AI	Adequate Intake
DRI	Dietary Reference Intake
OR	Odds Ratio
CI	Confidence Interval
SD	Standard Deviation
ROS	Reactive Oxygen Species
