# Supplementary material for: The micronutrient content of the diet is correlated with serum glucose biomarkers and lipid profile and is associated with the odds of being overweight/obese—a case-control study
Source: Front Nutr. 2023 Jun 29;10:1148183. doi: 10.3389/fnut.2023.1148183 (PMC10338876; doi:10.3389/fnut.2023.1148183)
Supplement: Supplementary file 1 [file Table_1.docx]

| **Supplementary Table 1.** Odds ratios (ORs) and 95% confidence intervals (CIs) for the association between INQ and BSA and BMI groups in men and women*. | | | | | | | | |
| --- | --- | --- | --- | --- | --- | --- | --- | --- |
| **INQs** | **BMI** | | | | **BSA** | | | |
|  | **Men (n= 798)** | | **Women (n= 807)** | | **Men (n= 798)** | | **Women (n= 807)** | |
|  | **ORs and CI 95%** | **P-value** | **ORs and CI 95%** | **P-value** | **ORs and CI 95%** | **P-value** | **ORs and CI 95%** | **P-value** |
| Vitamin A | **0.56 (0.35-0.91)** | **0.01** | 0.96 (0.58-1.59) | 0.88 | 1.05 (0.65-1.69) | 0.83 | 1.07 (0.63-1.80) | 0.79 |
| Vitamin D | 1.49 (0.24-9.27) | 0.66 | 0.43 (0.06-2.81) | 0.38 | 0.56 (0.08-3.90) | 0.56 | 0.37 (0.05-2.55) | 0.37 |
| Vitamin E | 0.82 (0.59-1.14) | 0.24 | 1.02 (0.72-1.46) | 0.88 | 0.89 (0.63-1.26) | 0.52 | 1.19 (0.82-1.73) | 0.34 |
| Vitamin K | **0.83 (0.71-0.95)** | **0.01** | 1.11 (0.96-1.27) | 0.14 | **0.85 (0.73-0.99)** | **0.04** | 0.97 (0.84-1.12) | 0.69 |
| Vitamin C | **0.62 (0.48-0.79)** | **<0.01** | 1.01 (0.80-1.27) | 0.92 | **0.72 (0.55-0.94)** | **0.01** | **0.89 (0.70-0.99)** | **0.05** |
| Thiamin | 0.87 (0.70-1.09) | 0.23 | 0.97 (0.77-1.24) | 0.85 | 1.03 (0.82-1.29) | 0.78 | 1.03 (0.80-1.32) | 0.79 |
| Riboflavin | 0.81 (0.63-1.06) | 0.14 | 0.98 (0.74-1.28) | 0.89 | 0.93 (0.71-1.23) | 0.64 | 0.98 (0.74-1.29) | 0.89 |
| Niacin | 0.84 (0.67-1.06) | 0.14 | 1.04 (0.81-1.34) | 0.71 | 0.88 (0.69-1.12) | 0.30 | 1.06 (0.81-1.38) | 0.63 |
| Vitamin B6 | 0.85 (0.63-1.15) | 0.30 | 1.14 (0.82-1.57) | 0.44 | 0.81 (0.58-1.11) | 0.20 | 1.15 (0.82-1.63) | 0.40 |
| Folate | 0.80 (0.60-1.06) | 0.14 | 0.94 (0.71-1.24) | 0.67 | 1.12 (0.84-1.50) | 0.42 | **0.75 (0.56-0.99)** | **0.04** |
| Vitamin B12 | 0.98 (0.85-1.14) | 0.88 | 0.92 (0.80-1.06) | 0.25 | 1.03 (0.89-1.20) | 0.64 | 0.98 (0.85-1.14) | 0.79 |
| Biotin | 0.73 (0.49-1.11) | 0.14 | 1.03 (0.67-1.57) | 0.88 | 1.02 (0.67-1.56) | 0.89 | 0.85 (0.55-1.32) | 0.48 |
| Pantothenic acid | 0.97 (0.71-1.32) | 0.86 | 0.83 (0.59-1.15) | 0.26 | 1.16 (0.84-1.61) | 0.34 | 0.79 (0.57-1.12) | 0.19 |
| Calcium | **0.53 (0.34-0.83)** | **<0.01** | **0.79 (0.50-0.98)** | **0.04** | 0.72 (0.45-1.15) | 0.17 | 0.86 (0.53-1.40) | 0.55 |
| Iron | 0.93 (0.74-1.16) | 0.52 | 0.87 (0.69-1.11) | 0.26 | 1.16 (0.92-1.46) | 0.20 | 1.01 (0.80-1.28) | 0.91 |
| Magnesium | 0.68 (0.45-1.03) | 0.06 | 0.82 (0.52-1.28) | 0.39 | 0.77 (0.50-1.19) | 0.24 | 0.65 (0.41-1.02) | 0.06 |
| Zinc | 0.78 (0.56-1.09) | 0.15 | 0.94 (0.67-1.32) | 0.74 | **0.66 (0.46-0.95)** | **0.02** | **0.74 (0.52-0.99)** | **0.05** |
| Copper | 0.94 (0.82-1.08) | 0.39 | 1.00 (0.87-1.14) | 0.97 | 0.90 (0.78-1.04) | 0.18 | 1.01 (0.88-1.16) | 0.84 |
| Selenium | **0.71 (0.58-0.88)** | **<0.01** | **0.82 (0.67-0.94)** | **0.05** | 0.91 (0.73-1.14) | 0.44 | 1.07 (0.86-1.33) | 0.52 |
| Manganese | 0.93 (0.84-1.03) | 0.20 | 0.98 (0.88-1.09) | 0.73 | 0.89 (0.79-1.00) | 0.06 | 0.98 (0.88-1.11) | 0.88 |
| * Logistic regression model adjusted for age, education, smoking, alcohol, marital status, regular physical activity, history of CVD, type 2 diabetes, and hypertension. Significant p-values are shown in **bold**.  INQ= Index of nutritional quality, BMI= Body Mass Index, BSA= Body Surface Area | | | | | | | | |

| **Supplementary Table 2.** Correlation between INQs and blood glucose handling markers and lipid profile. | | | | | | | | | | | | | | |
| --- | --- | --- | --- | --- | --- | --- | --- | --- | --- | --- | --- | --- | --- | --- |
| **INQs ^a, b^** | **FBS** | **P-value** | **Insulin** | **P-value** | **HbA1C** | **P-value** | **HDL-C** | **P-value** | **LDL-C** | **P-value** | **TC** | **P-value** | **TG** | **P-value** |
| Vitamin A | 0.022  0.019 | 0.19  0.23 | -0.039  -0.039 | 0.06  0.06 | -0.013  -0.012 | 0.31  0.31 | **0.054**  **0.055** | **0.02**  **0.01** | **-0.038**  -0.040 | **0.04**  0.06 | -0.016  -0.019 | 0.26  0.22 | -0.027  -0.026 | 0.14  0.15 |
| Vitamin D | 0.037  0.035 | 0.07  0.08 | **0.045**  **0.044** | **0.04**  **0.04** | 0.030  0.029 | 0.11  0.13 | 0.009  0.015 | 0.37  0.27 | 0.012  0.007 | 0.31  0.38 | 0.006  0.002 | 0.41  0.47 | 0.020  0.018 | 0.21  0.23 |
| Vitamin E | 0.014  0.010 | 0.28  0.34 | -0.020  -0.018 | 0.21  0.24 | -0.020  -0.021 | 0.21  0.20 | **0.043**  **0.047** | **0.04**  **0.03** | -0.011  -0.015 | 0.33  0.27 | -0.004  -0.007 | 0.44  0.38 | 0.026  0.028 | 0.15  0.13 |
| Vitamin K | 0.018  0.023 | 0.24  0.18 | **-0.060**  **-0.058** | **<0.01**  **0.01** | **0.050**  **0.051** | **0.02**  **0.02** | 0.023  0.022 | 0.18  0.19 | 0.038  **0.042** | 0.06  **0.05** | 0.018  0.020 | 0.23  0.22 | -0.026  -0.026 | 0.15  0.15 |
| Vitamin C | -0.010  -0.006 | 0.35  0.40 | -0.002  -0.005 | 0.46  0.42 | 0.002  0.003 | 0.47  0.45 | 0.018  0.016 | 0.23  0.26 | -0.014  -0.012 | 0.28  0.32 | -0.020  -0.018 | 0.21  0.23 | 0.016  0.017 | 0.26  0.25 |
| Thiamin | -0.010  -0.012 | 0.34  0.31 | -0.003  -0.003 | 0.45  0.45 | 0.020  0.020 | 0.21  0.22 | 0.030  0.034 | 0.11  0.09 | 0.005  0.003 | 0.43  0.45 | 0.004  0.004 | 0.43  0.44 | **-0.041**  **-0.041** | **0.05**  **0.05** |
| Riboflavin | -0.006  -0.008 | 0.40  0.37 | 0.022  0.024 | 0.18  0.17 | -0.016  -0.017 | 0.26  0.25 | **0.060**  **0.061** | **<0.01**  **<0.01** | -0.006  -0.011 | 0.40  0.33 | -0.022  -0.025 | 0.19  0.16 | -0.035  -0.036 | 0.08  0.07 |
| Niacin | 0.006  0.006 | 0.40  0.39 | 0.021  0.024 | 0.20  0.17 | -0.005  -0.005 | 0.42  0.42 | 0.029  0.028 | 0.13  0.13 | -0.009  -0.010 | 0.36  0.35 | 0.005  0.003 | 0.43  0.45 | 0.017  0.017 | 0.25  0.25 |
| Vitamin B6 | **0.056**  **0.052** | **0.02**  **0.02** | -0.010  -0.007 | 0.34  0.39 | 0.038  0.038 | 0.07  0.06 | 0.034  0.037 | 0.08  0.07 | 0.004  0.001 | 0.44  0.49 | 0.010  0.007 | 0.35  0.39 | -0.005  -0.005 | 0.41  0.41 |
| Folate | 0.005  0.005 | 0.41  0.42 | 0.018  0.018 | 0.24  0.24 | 0.003  0.002 | 0.46  0.46 | **0.061**  **0.062** | **<0.01**  **<0.01** | 0.008  0.007 | 0.37  0.38 | 0.005  0.004 | 0.43  0.43 | -0.002  -0.002 | 0.46  0.46 |
| Vitamin B12 | 0.030  0.029 | 0.12  0.12 | 0.030  0.030 | 0.11  0.12 | -0.028  -0.028 | 0.13  0.13 | 0.003  0.002 | 0.45  0.47 | -0.010  -0.009 | 0.34  0.35 | -0.006  -0.006 | 0.41  0.41 | 0.021  0.022 | 0.20  0.19 |
| Biotin | 0.026  0.027 | 0.15  0.14 | 0.001  0.003 | 0.50  0.46 | -0.023  -0.024 | 0.17  0.17 | 0.031  0.034 | 0.10  0.08 | 0.035  0.033 | 0.08  0.10 | **0.047**  **0.045** | **0.03**  **0.04** | 0.003  0.002 | 0.45  0.47 |
| Pantothenic acid | 0.013  0.011 | 0.29  0.33 | 0.027  0.028 | 0.14  0.13 | -0.018  -0.019 | 0.24  0.23 | 0.005  0.009 | 0.41  0.36 | 0.001  -0.004 | 0.49  0.43 | -0.017  -0.020 | 0.25  0.21 | 0.002  0.002 | 0.46  0.47 |
| Calcium | **-0.046**  **-0.046** | **0.03**  **0.03** | 0.008  0.011 | 0.37  0.33 | 0.024  0.025 | 0.16  0.16 | **0.045**  **0.047** | **0.04**  **0.03** | **-0.042**  **-0.045** | **0.04**  **0.03** | -0.016  -0.018 | 0.26  0.23 | -0.003  -0.003 | 0.45  0.044 |
| Iron | 0.019  0.021 | 0.22  0.19 | 0.025  0.024 | 0.16  0.17 | -0.031  -0.031 | 0.11  0.11 | **0.047**  **0.047** | **0.03**  **0.03** | 0.003  0.003 | 0.45  0.45 | -0.004  -0.004 | 0.44  0.43 | -0.021  -0.021 | 0.20  0.20 |
| Magnesium | -0.001  -0.001 | 0.48  0.48 | 0.003  0.003 | 0.45  0.46 | **-0.052**  **-0.051** | **0.02**  **0.02** | 0.019  0.021 | 0.22  0.20 | **-0.041**  **-0.042** | **0.05**  **0.05** | **-0.042**  **-0.044** | **0.04**  **0.04** | **-0.042**  **-0.042** | **0.05**  **0.05** |
| Zinc | -0.012  -0.012 | 0.31  0.31 | -0.004  -0.005 | 0.43  0.41 | -0.031  -0.032 | 0.11  0.10 | **0.042**  **0.042** | **0.05**  **0.05** | 0.011  0.009 | 0.32  0.36 | 0.006  0.006 | 0.40  0.41 | -0.008  -0.009 | 0.38  0.36 |
| Copper | -0.017  -0.015 | 0.25  0.27 | -0.009  -0.008 | 0.35  0.37 | -0.004  -0.002 | 0.44  0.46 | 0.022  0.021 | 0.19  0.20 | 0.018  0.019 | 0.23  0.22 | 0.016  0.016 | 0.26  0.26 | -0.016  -0.016 | 0.26  0.26 |
| Selenium | -0.021  -0.023 | 0.20  0.18 | 0.004  0.007 | 0.43  0.38 | -0.012  -0.014 | 0.31  0.28 | -0.008  -0.002 | 0.37  0.47 | 0.013  0.004 | 0.30  0.43 | 0.014  0.008 | 0.29  0.37 | -0.003  -0.005 | 0.45  0.41 |
| Manganese | 0.010  0.010 | 0.35  0.35 | 0.007  0.007 | 0.40  0.38 | -0.012  -0.013 | 0.31  0.30 | **0.049**  **0.049** | **0.02**  **0.02** | -0.020  -0.021 | 0.21  0.20 | -0.016  -0.015 | 0.26  0.27 | 0.001  0.001 | 0.48  0.48 |
| ^a^ Bivariate correlation  ^b^ Partial correlation controlling for age, gender, education, smoking, alcohol, marital status, regular physical activity, history of CVD, type 2 diabetes, and hypertension.  INQ= Index of nutritional quality, FBS= Fasting blood sugar, HbA1C= Glycated hemoglobin, LDL-C= Low-density lipoprotein-cholesterol, TC= Total cholesterol, TG= Triglycerides, HDL-C= High-density lipoprotein-cholesterol.  Significant p-values are shown in **bold**. | | | | | | | | | | | | | | |
